# Supplementary material for: Presenting native-like trimeric HIV-1 antigens with self-assembling nanoparticles
Source: Nat Commun. 2016 Jun 28;7:12041. doi: 10.1038/ncomms12041 (PMC4931238; doi:10.1038/ncomms12041)
Supplement: Supplementary Information — Supplementary Figures 1-7 and Supplementary Table 1 [file ncomms12041-s1.pdf]

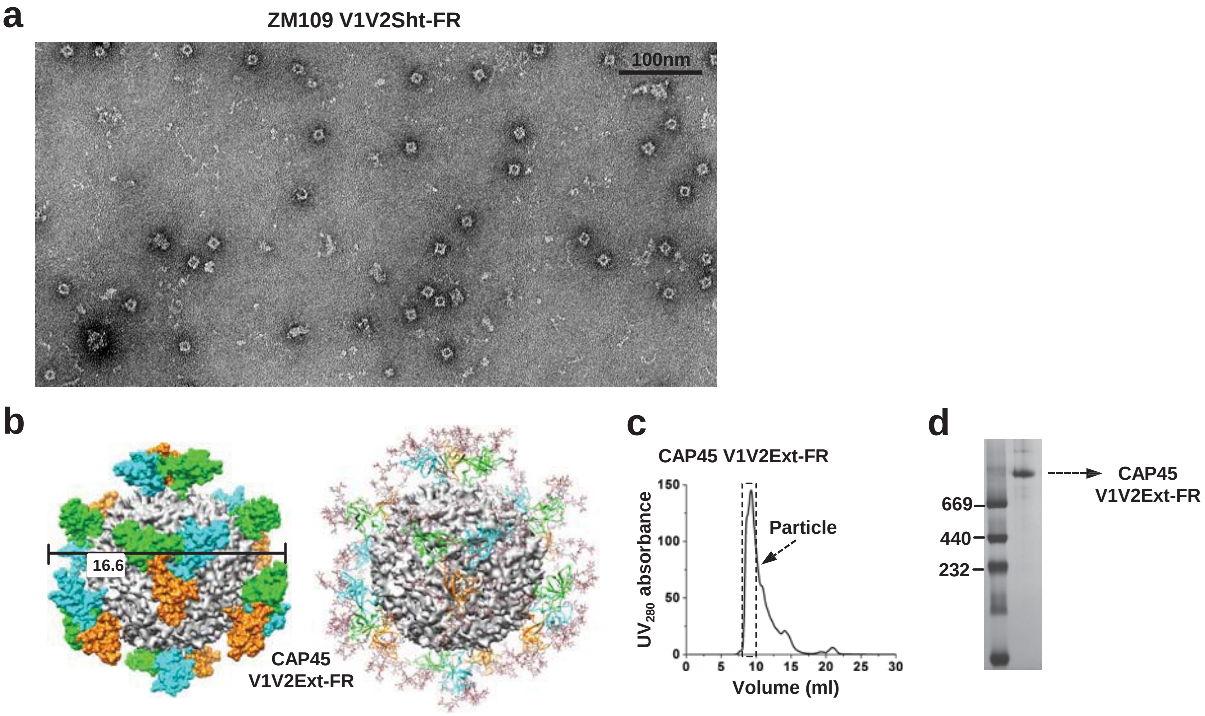

Supplementary Figure 1. V1V2-ferritin nanoparticles. (**a**) Micrograph of ZM109 V1V2Sht-FR derived from negative-stain EM. (**b**) Structural model of V1V2Ext-FR nanoparticle designed based on the V1V2 of clade-C CAP45. The surface model of protein particle is shown on the left while the ribbon model of trimeric V1V2 spikes decorated with *N*-linked glycans is shown on the right. Ferritin and three V1V2 chains within each trimeric spike are colored in gray, cyan, green, and orange, respectively. (**c**) SEC profile of CAP45 V1V2Ext-FR from a Superdex 200 10/300 GL column. Fractions used for BN-PAGE (8.25-10.0 ml) are indicated with a dashed box. (**d**) BN-PAGE of CAP45 V1V2Ext-FR, with a band of high molecular weight corresponding to well-formed nanoparticles.

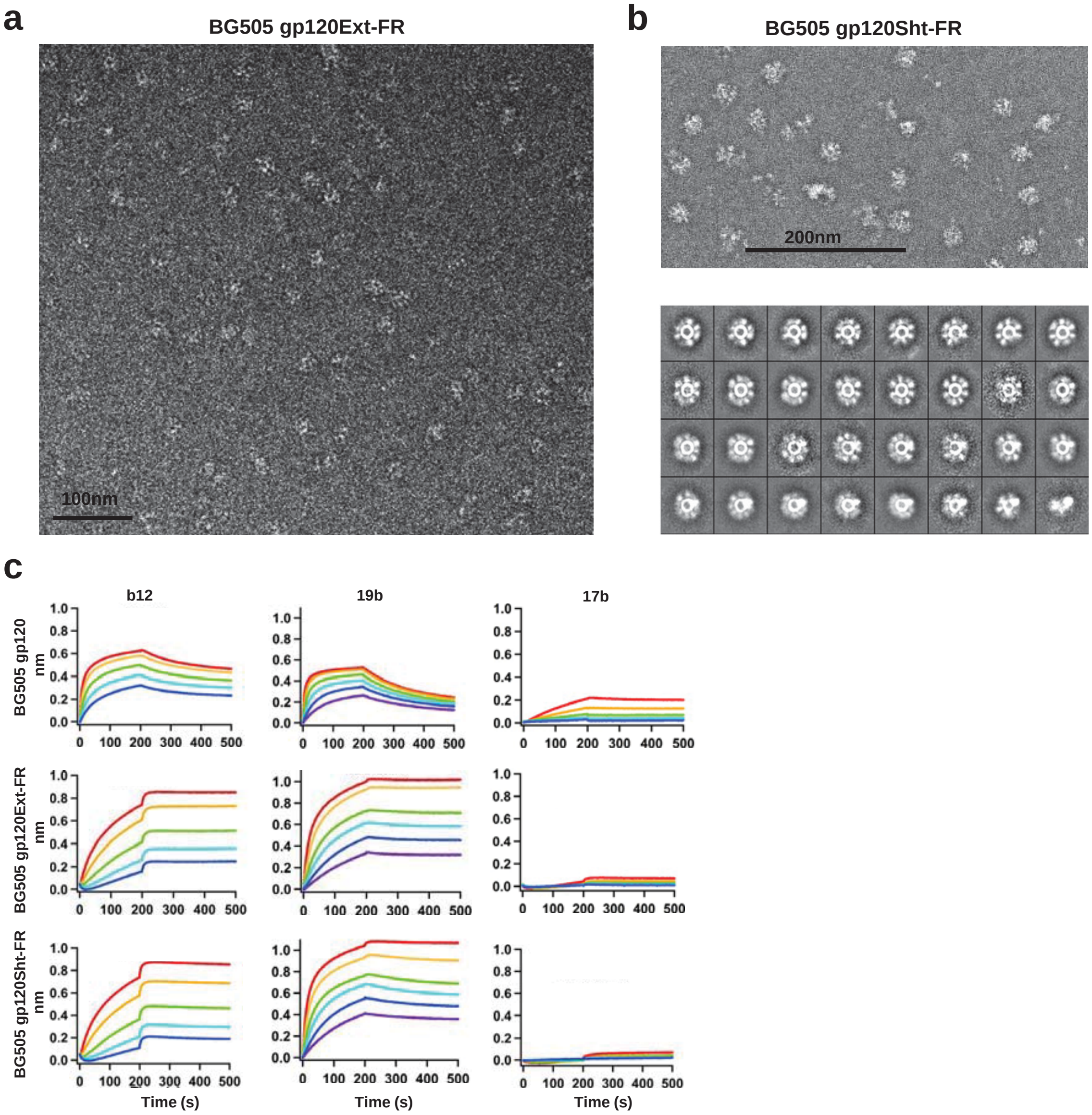

Supplementary Figure 2. BG505 gp120-ferritin nanoparticles. (**a**) Micrograph of gp120Ext-FR derived from negative-stain EM. (**b**) Micrograph (upper panel) and 2D class averages (lower panel) of gp120Sht-FR derived from negative-stain EM. (**c**) Octet binding of monomeric gp120 and two gp120-FR nanoparticles to the CD4bs-directed NAb b12 and two non-NAbs (V3-specific 19b and CD4i-specific 17b). Sensorgrams were obtained from an Octet RED96 instrument using a titration series of six concentrations starting at the maximum of 200 nM for monomeric gp120 and 50 nM for two gp120-FR nanoparticles, respectively.

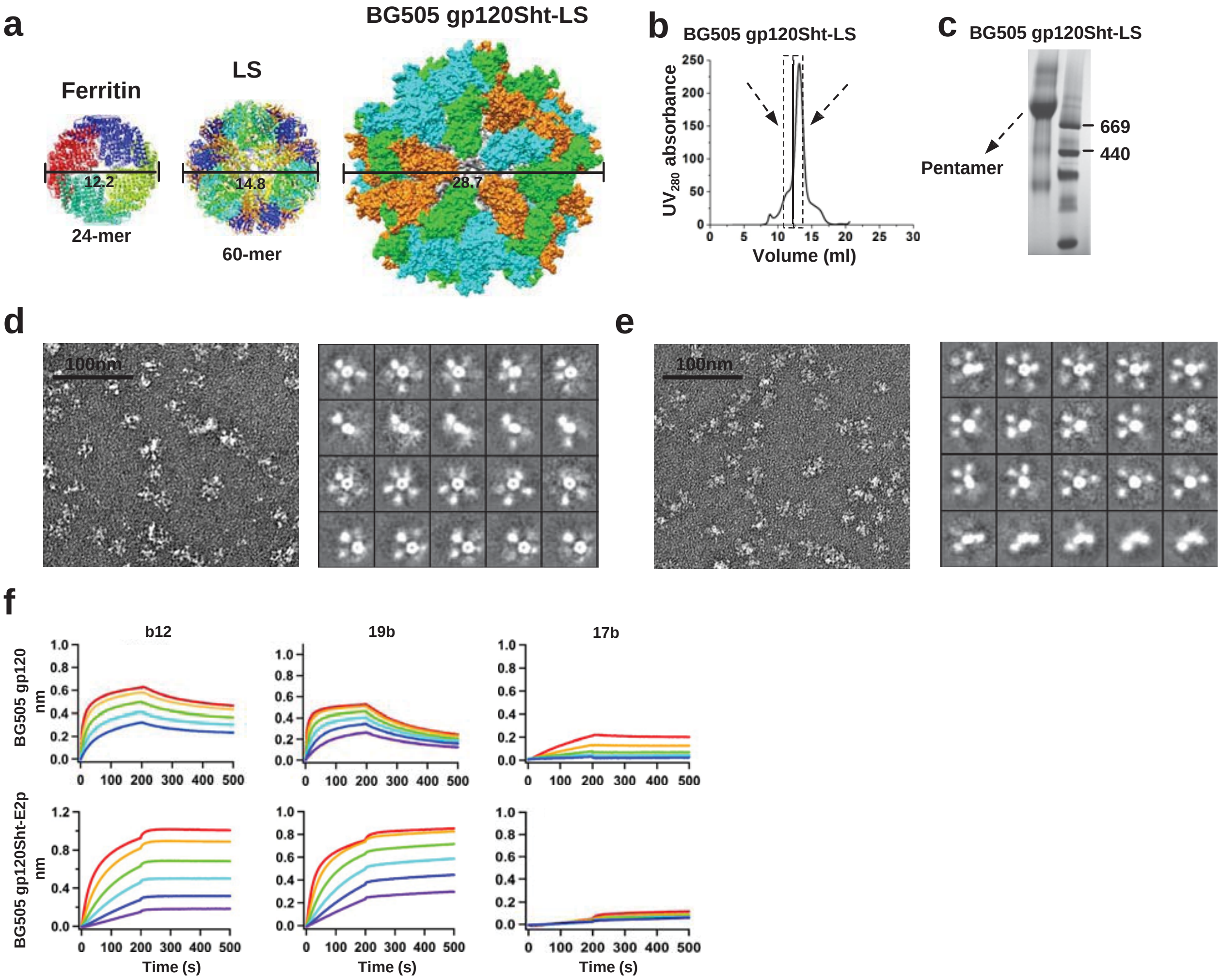

Supplementary Figure 3. BG505 gp120 nanoparticles based on the LS and E2p 60-mers. (**a**) Structural models of ferritin, LS, and gp120Sht-LS nanoparticles. The ribbon models of ferritin and LS are color-coded based on protein chains. For gp120Sht-LS, LS and three gp120 chains within each trimeric spike are colored in gray, cyan, green, and orange, respectively. (**b**) SEC profile of gp120Sht-LS from a Superose 6 10/300 GL column. Two fractions used for EM are indicated with dashed boxes. (**c**) BN-PAGE of gp120Sht-LS. (**d**) and (**e**) Micrograph (left) and 2D class averages (right) of gp120Sht-LS derived from negative-stain EM for SEC fractions 11.25-12.5 and 12.5-14.0 ml, respectively. (**f**) Octet binding of monomeric gp120 and gp120Sht-E2p nanoparticle to the CD4bs-directed NAb b12 and two non-NAbs (V3-specific 19b and CD4i-specific 17b). Sensorgrams were obtained from an Octet RED96 instrument using a titration series of six concentrations starting at the maximum of 200 nM for monomeric gp120 and 15 nM for gp120Sht-E2p nanoparticle, respectively.

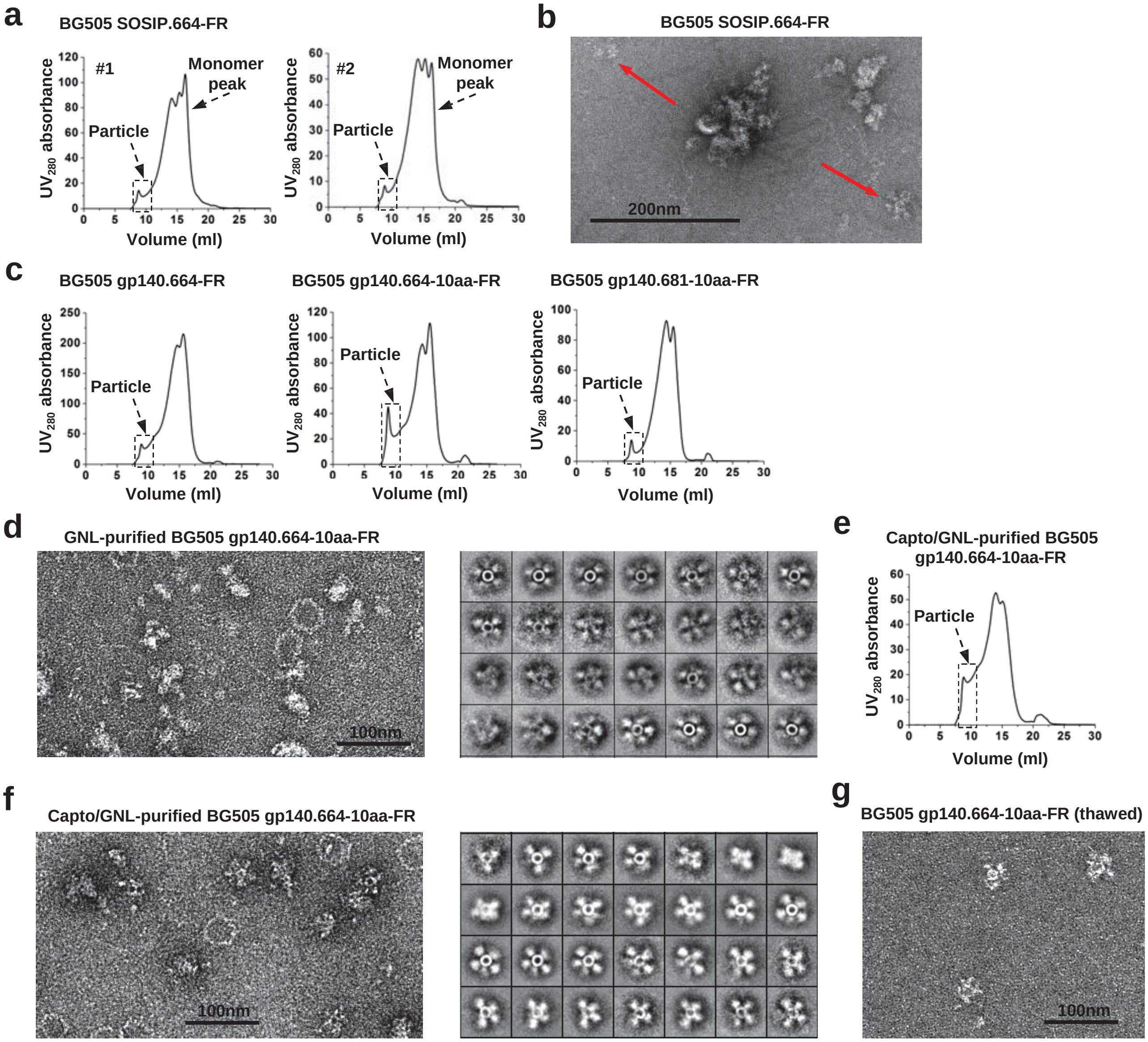

Supplementary Figure 4. SEC and EM analyses of BG505 gp140-ferritin nanoparticles. (**a**) SEC profiles of GNL-purified SOSIP-FR from a Superose 6 10/300 GL column. Fractions used for EM (8.0-10.75 ml) are indicated with a dashed box, with the misfolded monomer peak also labeled for comparison. (**b**) Micrograph of GNL-purified SOSIP-FR derived from negative-stain EM. Two particles are indicated with red arrows. (**c**) SEC profiles of GNL-purified gp140.664-FR, gp140.664-10aa-FR, and gp140.681-10aa-FR from a Superose 6 10/300 GL column. Fractions used for EM (8.0-10.75 ml) are indicated with a dashed box. (**d**) Micrograph (left) and 2D class averages (right) of GNL-purified gp140.664-10aa-FR derived from negative-stain EM. (**e**) SEC profile of Capto/GNL-purified gp140.664-10aa-FR from a Superose 6 10/300 GL column. Fractions used for EM (8.0-10.75 ml) are indicated with a dashed box. (**f**) Micrograph (left) and 2D class averages (right) of Capto/GNL-purified gp140.664-10aa-FR derived from negative-stain EM. (**g**) Micrograph of a gp140.664-10aa-FR sample that has been frozen and thawed, derived from negative-stain EM.

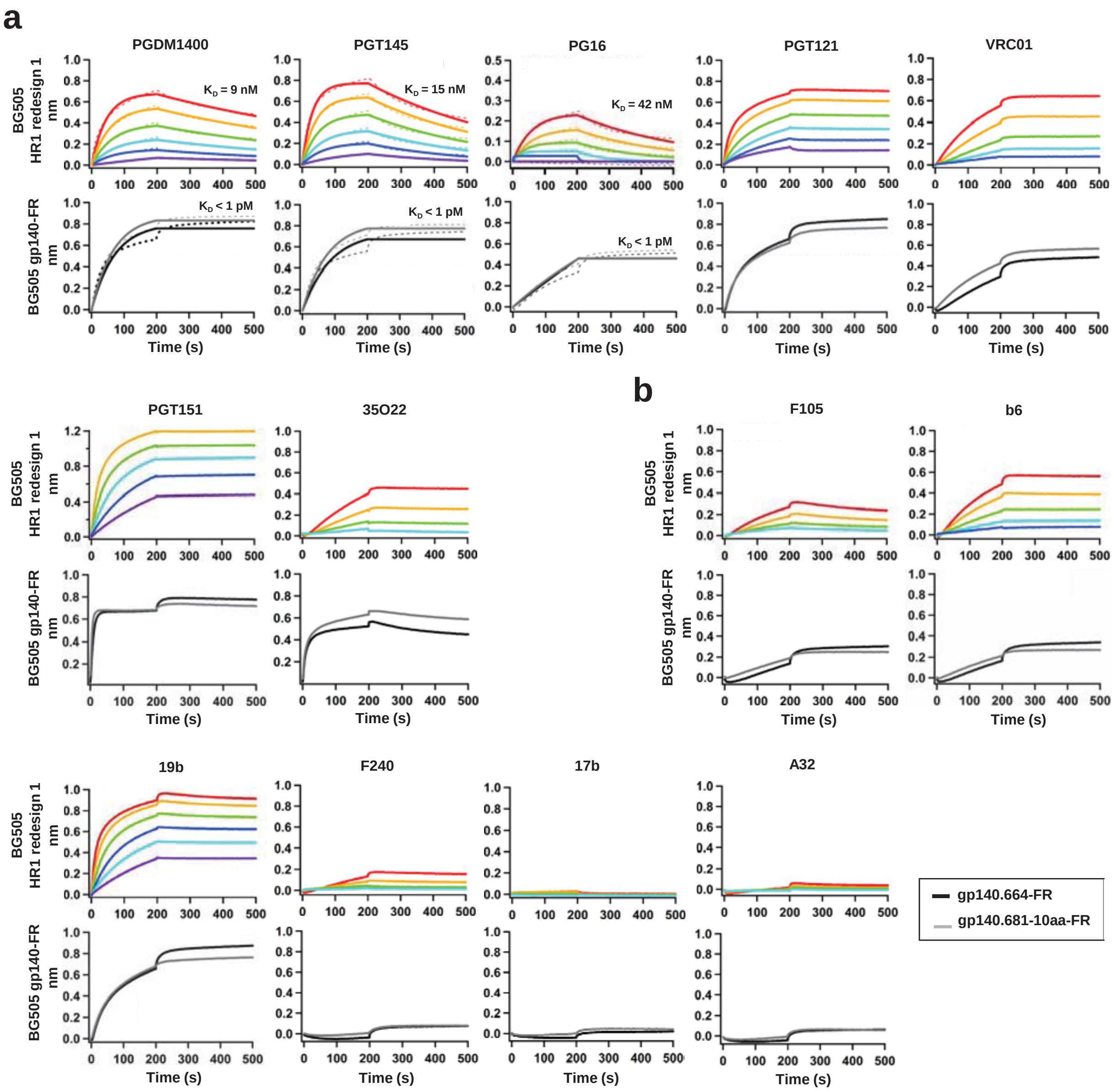

Supplementary Figure 5. Antigenicity of BG505 gp140-ferritin nanoparticles. Octet binding of HR1-redesigned gp140 trimer and two gp140-FR nanoparticles to a panel of (**a**) bNAbs and (**b**) non-NAbs. Sensorgrams were obtained from an Octet RED96 instrument using a titration series of six concentrations starting at the maximum of 200 nM for the trimer and a single concentration at 35 nM for gp140.664-FR and gp140.681-10aa-FR, respectively. K_D values are calculated from 1:1 global fitting for apex-directed bNAbs PGDM1400, PGT145, and PG16 in (a). Sensorgrams are represented by black and grey lines for gp140.664-FR and gp140.681-10aa-FR, respectively.

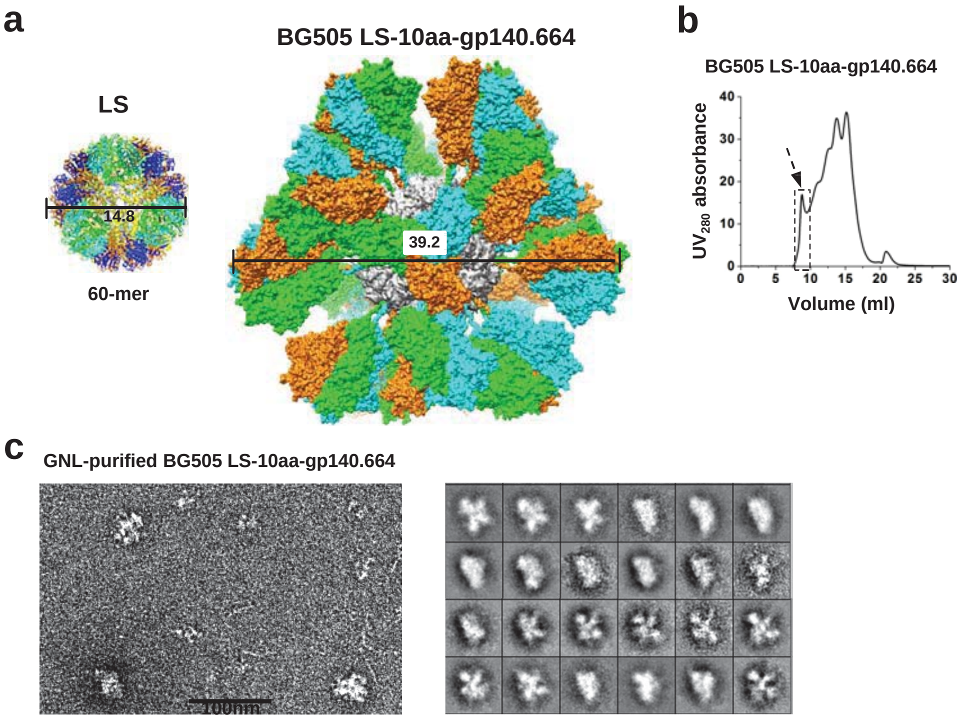

Supplementary Figure 6. Design and characterization of BG505 gp140 nanoparticle based on 60-meric LS. (**a**) Structural models of LS and LS-10aa-gp140.664 nanoparticle. The ribbon model of LS is color-coded based on protein chains. For LS-10aa-gp140.664, LS and three gp140 chains within each Env trimer are colored in gray, cyan, green, and orange, respectively. (**b**) SEC profile of LS-10aa-gp140.664 from a Superose 6 10/300 GL column. Fractions used for EM (8.0-10.0 ml) are indicated with a dashed box. (**c**) Micrograph (left) and 2D class averages (right) of LS-10aa-gp140.664 derived from negative-stain EM.

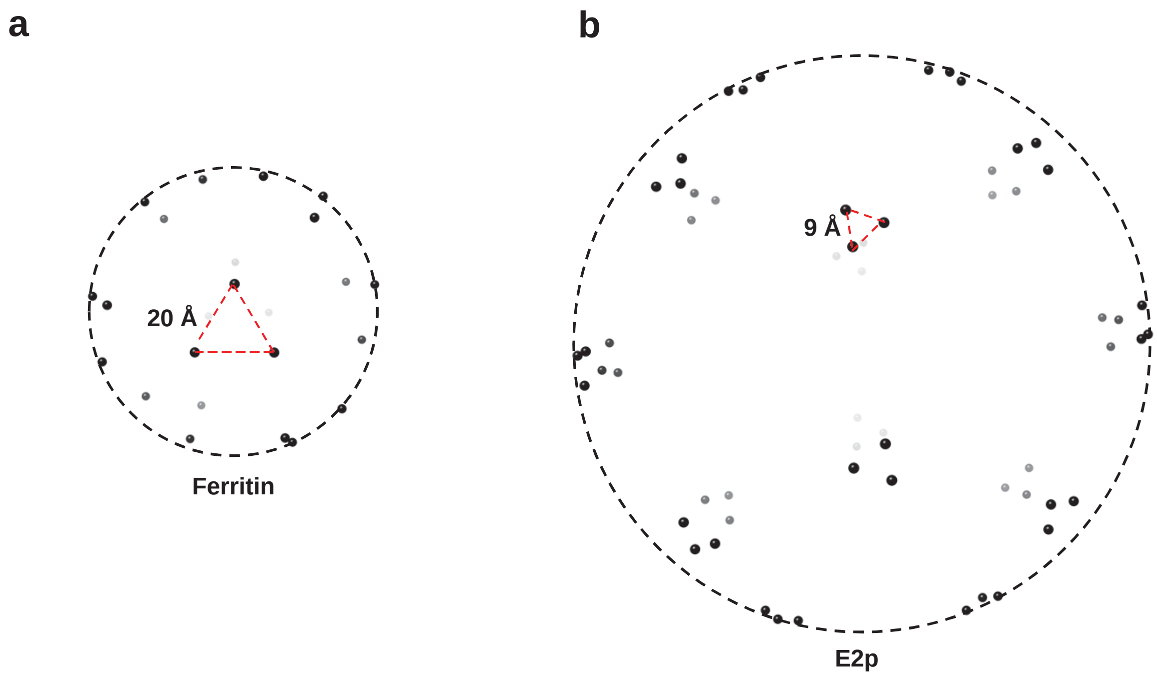

Supplementary Figure 7. Mapping of gp140-fusion sites on ferritin and E2p nanoparticles. (**a**) Eight 3-fold gp140-fusion sites on ferritin surface defined by the N-terminal residue Asp5 of 24 ferritin subunits. (**b**) Twenty 3-fold gp140-fusion sites on E2p surface defined by the N-terminal residue Ala185 of 60 E2p subunits. The edge within each 3-fold gp140-fusion site is 20 Å and 9 Å for ferritin and E2p, respectively.

Table S1. Amino acid sequences of HIV-1 trimer-presenting nanoparticles.[a]

Construct name	Amino acid sequence
a. V1V2 trimer-presenting ferritin nanoparticles	
ZM109 V1V2Ext-FR	PCVKLTPLCVTLNCTSPAAHNESETRVKHCSFNITTDVKDRKQKVNATFYDLDIVPLSSSDNSSN<br>SSLYRLISCNTSTITQACPASGDIIKLLNEQVNKEMQSSNLYMSMSSWCYTHSLDGAGLFLFDHA<br>AEEYEHAKKLIIFLNENNVPVQLTSISAPEHKFEGLTQIFQKAYEHEQHISESINNIVDHAIKSKDH<br>ATFNFLQWYVAEQHEEEVLFKDILDKIELIGNENHGLYLADQYVKGIAKSRKS
ZM109 V1V2Sht-FR	ACVTLNCTSPAAHNESETRVKHCSFNITTDVKDRKQKVNATFYDLDIVPLSSSDNSSNSSLYRLI<br>SCAASGDIIKLLNEQVNKEMQSSNLYMSMSSWCYTHSLDGAGLFLFDHAAEEYEHAKKLIIFLN<br>ENNVPVQLTSISAPEHKFEGLTQIFQKAYEHEQHISESINNIVDHAIKSKDHATFNFLQWYVAEQ<br>HEEEVLFKDILDKIELIGNENHGLYLADQYVKGIAKSRKS
CAP45 V1V2Ext-FR	PCVKLTPLCVTLRCTNATINGSLTEEVKNCSFNITTELRDKKQKAYALFYRPDVVPLNKNSPSGN<br>SSEYILINCNTSTITQACPASGDIIKLLNEQVNKEMQSSNLYMSMSSWCYTHSLDGAGLFLFDHA<br>AEEYEHAKKLIIFLNENNVPVQLTSISAPEHKFEGLTQIFQKAYEHEQHISESINNIVDHAIKSKDH<br>ATFNFLQWYVAEQHEEEVLFKDILDKIELIGNENHGLYLADQYVKGIAKSRKS
b. gp120 trimer-presenting ferritin nanoparticles	
BG505 gp120Ext-FR	GVPVWKDAETTLFCASDAKAYDTEKHNVWATHACVPTDPNPQEIHLENVTEEFNMWKNNMV<br>EQMHTDIISLWDQSLKPCVKLTPLCVTLQCTNVTNNITDDMRGELKNCSFNMTTELRDKKQKV<br>YSLFYRLDVVQINENQGNRSNNSNKEYRLINCNTSAITQACPKVSFEPIPIHYCAPAGFAILKCKD<br>KKFNGTGPCPSVSTVQCTHGIKPVVSTQLLLNGSLAEEEVMIRSENITNNAKNILVQFNTPVQIN<br>CTRPNNNTRKSIRIGPGQAFYATGDIIGDIRQAHCNVSKATWNETLGKVVKQLRKHFGNNTIIRF<br>ANSSGGDLEVTTHSFNCGGEFFYCNTSGLFNSTWISNTSVQGSNSTGSNDSITLPCRIKQIINMW<br>QRIGQAMYAPPIQGVIRCVSNITGLILTRDGGSTNSTTETFRPGGGDMRDNWRSELYKYKVVKI<br>EPLGASGDIIKLLNEQVNKEMQSSNLYMSMSSWCYTHSLDGAGLFLFDHAAEEYEHAKKLIIFL<br>NENNVPVQLTSISAPEHKFEGLTQIFQKAYEHEQHISESINNIVDHAIKSKDHATFNFLQWYVAE<br>QHEEEVLFKDILDKIELIGNENHGLYLADQYVKGIAKSRKS
BG505 gp120Sht-FR	GVWKDAETTLFCASDAKAYDTEKHNVWATHACVPTDPNPQEIHLENVTEEFNMWKNNMVEQ<br>MHTDIISLWDQSLKPCVKLTPLCVTLQCTNVTNNITDDMRGELKNCSFNMTTELRDKKQKVYS<br>LFYRLDVVQINENQGNRSNNSNKEYRLINCNTSAITQACPKVSFEPIPIHYCAPAGFAILKCKDK<br>KFNGTGPCPSVSTVQCTHGIKPVVSTQLLLNGSLAEEEVMIRSENITNNAKNILVQFNTPVQINCT<br>RPNNNTRKSIRIGPGQAFYATGDIIGDIRQAHCNVSKATWNETLGKVVKQLRKHFGNNTIIRFAN<br>SSGGDLEVTTHSFNCGGEFFYCNTSGLFNSTWISNTSVQGSNSTGSNDSITLPCRIKQIINMWQRI<br>GQAMYAPPIQGVIRCVSNITGLILTRDGGSTNSTTETFRPGGGDMRDNWRSELYKYKVVKIEGA<br>SGDIIKLLNEQVNKEMQSSNLYMSMSSWCYTHSLDGAGLFLFDHAAEEYEHAKKLIIFLNENNV<br>PVQLTSISAPEHKFEGLTQIFQKAYEHEQHISESINNIVDHAIKSKDHATFNFLQWYVAEQHEEEV<br>LFKDILDKIELIGNENHGLYLADQYVKGIAKSRKS
BG505 gp120SS-FR	GWCDAETTLFCASDAKAYDTEKHNVWATHACVPTDPNPQEIHLENVTEEFNMWKNNMVEQM<br>HTDIISLWDQSLKPCVKLTPLCVTLQCTNVTNNITDDMRGELKNCSFNMTTELRDKKQKVYSLF<br>YRLDVVQINENQGNRSNNSNKEYRLINCNTSAITQACPKVSFEPIPIHYCAPAGFAILKCKDKKF<br>NGTGPCPSVSTVQCTHGIKPVVSTQLLLNGSLAEEEVMIRSENITNNAKNILVQFNTPVQINCTRP<br>NNNTRKSIRIGPGQAFYATGDIIGDIRQAHCNVSKATWNETLGKVVKQLRKHFGNNTIIRFANSS<br>GGDLEVTTHSFNCGGEFFYCNTSGLFNSTWISNTSVQGSNSTGSNDSITLPCRIKQIINMWQRIGQ<br>AMYAPPIQGVIRCVSNITGLILTRDGGSTNSTTETFRPGGGDMRDNWRSELYKYKVVCIGASGDI<br>IKLLNEQVNKEMQSSNLYMSMSSWCYTHSLDGAGLFLFDHAAEEYEHAKKLIIFLNENNVPVQ<br>LTSISAPEHKFEGLTQIFQKAYEHEQHISESINNIVDHAIKSKDHATFNFLQWYVAEQHEEEVLFK<br>DILDKIELIGNENHGLYLADQYVKGIAKSRKS
c. gp120 trimer-presenting LS and E2p nanoparticles	
BG505 gp120Sht-LS	GVWKDAETTLFCASDAKAYDTEKHNVWATHACVPTDPNPQEIHLENVTEEFNMWKNNMVEQ<br>MHTDIISLWDQSLKPCVKLTPLCVTLQCTNVTNNITDDMRGELKNCSFNMTTELRDKKQKVYS<br>LFYRLDVVQINENQGNRSNNSNKEYRLINCNTSAITQACPKVSFEPIPIHYCAPAGFAILKCKDK<br>KFNGTGPCPSVSTVQCTHGIKPVVSTQLLLNGSLAEEEVMIRSENITNNAKNILVQFNTPVQINCT<br>RPNNNTRKSIRIGPGQAFYATGDIIGDIRQAHCNVSKATWNETLGKVVKQLRKHFGNNTIIRFAN<br>SSGGDLEVTTHSFNCGGEFFYCNTSGLFNSTWISNTSVQGSNSTGSNDSITLPCRIKQIINMWQRI<br>GQAMYAPPIQGVIRCVSNITGLILTRDGGSTNSTTETFRPGGGDMRDNWRSELYKYKVVKIEGA<br>SGMQIYEGKLTAEGLRFGIVASRFNHALVDRLVEGAIDCIVRHGGREEDITLVRVPGSWEIPVAA<br>GELARKEDIDAVIAIGVLIRGATPHFDYIASEVSKGLANLALELRKPITFGVITADTLEQAIERAGT<br>KHGNKGWEAALSAIEMANLFKSLR

BG505 gp120Sht-E2p	GVWKDAETTLFCASDAKAYDTEKHNVWATHACVPTDPNPQEIHLENVTEEFNMWKNNMVEQ<br>MHTDIISLWDQSLKPCVKLTPLCVTLQCTNVTNNITDDMRGELKNCSFNMTTELRDKKQKVYS<br>LFYRLDVVQINENQGNRSNNSNKEYRLINCNTSAITQACPKVSFEPIPIHYCAPAGFAILKCKDK<br>KFNGTGPCPSVSTVQCTHGIKPVVSTQLLLNGSLAEEEVMIRSENITNNAKNILVQFNTPVQINCT<br>RPNNNTRKSIRIGPGQAFYATGDIIGDIRQAHCNVSKATWNETLGKVVKQLRKHFGNNTIIRFAN<br>SSGGDLEVTTHSFNCGGEFFYCNTSGLFNSTWISNTSVQGSNSTGSNDSITLPCRIKQIINMWQRI<br>GQAMYAPPIQGVIRCVSNITGLILTRDGGSTNSTTETFRPGGGDMRDNWRSELYKYKVVKIEGA<br>SGAAAKPATTEGEFPETREKMSGIRRAIAKAMVHSKHTAPHVTLMDEADVTKLVAHRKKFKAI<br>AAEKGIKLTFLPYVVKALVSALREYPVLNTAIDDETEEIIQKHYYNIGIAADTDRGLLVPVIKHA<br>DRKPIFALAQEINELAEKARDGKLTPGEMKGASCTITNIGSAGGQWFTPVINHPEVAILGIGRIAE<br>KPIVRDGEIVAAPMLALSLSFDHRMIDGATAQKALNHIKRLLSDPELLLM

d. gp140 trimer-presenting ferritin nanoparticles[b]

BG505 gp140.664-FR	AENLWVTVYYGVPVWKDAETTLFCASDAKAYETEKHNVWATHACVPTDPNPQEIHLENVTEE<br>FNMWKNNMVEQMHTDIISLWDQSLKPCVKLTPLCVTLQCTNVTNNITDDMRGELKNCSFNMT<br>TELRDKKQKVYSLFYRLDVVQINENQGNRSNNSNKEYRLINCNTSAITQACPKVSFEPIPIHYCA<br>PAGFAILKCKDKKFNGTGPCPSVSTVQCTHGIKPVVSTQLLLNGSLAEEEVMIRSENITNNAKNI<br>LVQFNTPVQINCTRPNNNTRKSIRIGPGQAFYATGDIIGDIRQAHCNVSKATWNETLGKVVKQL<br>RKHFGNNTIIRFANSSGGDLEVTTHSFNCGGEFFYCNTSGLFNSTWISNTSVQGSNSTGSNDSITL<br>PCRIKQIINMWQRIGQAMYAPPIQGVIRCVSNITGLILTRDGGSTNSTTETFRPGGGDMRDNWRS<br>ELYKYKVVKIEPLGVAPTRCKRRVVGRRRRRRAVGIGAVFLGFLGAAGSTMGAASMTLTVQA<br>RNLLSGNPDWLPDMTVWGIKQLQARVLAVERYLRDQQLLGIWGCSGKLICCTNVPWNSSWSN<br>RNLSEIWDNMTWLQWDKEISNYTQIIYGLLEESQNQQEKNEQDLLALD ASGDIIKLLNEQVNKE<br>MQSSNLYMSMSSWCYTHSLDGAGLFLFDHAAEEYEHAKKLIIFLNENNVPVQLTSISAPEHKFE<br>GLTQIFQKAYEHEQHISESINNIVDHAIKSKDHATFNFLQWYVAEQHEEEVLFKDILDKIELIGNE<br>NHGLYLADQYVKGIAKSRKS
BG505 gp140.664-10aa-FR	AENLWVTVYYGVPVWKDAETTLFCASDAKAYETEKHNVWATHACVPTDPNPQEIHLENVTEE<br>FNMWKNNMVEQMHTDIISLWDQSLKPCVKLTPLCVTLQCTNVTNNITDDMRGELKNCSFNMT<br>TELRDKKQKVYSLFYRLDVVQINENQGNRSNNSNKEYRLINCNTSAITQACPKVSFEPIPIHYCA<br>PAGFAILKCKDKKFNGTGPCPSVSTVQCTHGIKPVVSTQLLLNGSLAEEEVMIRSENITNNAKNI<br>LVQFNTPVQINCTRPNNNTRKSIRIGPGQAFYATGDIIGDIRQAHCNVSKATWNETLGKVVKQL<br>RKHFGNNTIIRFANSSGGDLEVTTHSFNCGGEFFYCNTSGLFNSTWISNTSVQGSNSTGSNDSITL<br>PCRIKQIINMWQRIGQAMYAPPIQGVIRCVSNITGLILTRDGGSTNSTTETFRPGGGDMRDNWRS<br>ELYKYKVVKIEPLGVAPTRCKRRVVGRRRRRRAVGIGAVFLGFLGAAGSTMGAASMTLTVQA<br>RNLLSGNPDWLPDMTVWGIKQLQARVLAVERYLRDQQLLGIWGCSGKLICCTNVPWNSSWSN<br>RNLSEIWDNMTWLQWDKEISNYTQIIYGLLEESQNQQEKNEQDLLALDGSGSGSGSGSASGDII<br>KLLNEQVNKEMQSSNLYMSMSSWCYTHSLDGAGLFLFDHAAEEYEHAKKLIIFLNENNVPVQL<br>TSISAPEHKFEGLTQIFQKAYEHEQHISESINNIVDHAIKSKDHATFNFLQWYVAEQHEEEVLFKD<br>ILDKIELIGNENHGLYLADQYVKGIAKSRKS
BG505 gp140.681-10aa-FR	AENLWVTVYYGVPVWKDAETTLFCASDAKAYETEKHNVWATHACVPTDPNPQEIHLENVTEE<br>FNMWKNNMVEQMHTDIISLWDQSLKPCVKLTPLCVTLQCTNVTNNITDDMRGELKNCSFNMT<br>TELRDKKQKVYSLFYRLDVVQINENQGNRSNNSNKEYRLINCNTSAITQACPKVSFEPIPIHYCA<br>PAGFAILKCKDKKFNGTGPCPSVSTVQCTHGIKPVVSTQLLLNGSLAEEEVMIRSENITNNAKNI<br>LVQFNTPVQINCTRPNNNTRKSIRIGPGQAFYATGDIIGDIRQAHCNVSKATWNETLGKVVKQL<br>RKHFGNNTIIRFANSSGGDLEVTTHSFNCGGEFFYCNTSGLFNSTWISNTSVQGSNSTGSNDSITL<br>PCRIKQIINMWQRIGQAMYAPPIQGVIRCVSNITGLILTRDGGSTNSTTETFRPGGGDMRDNWRS<br>ELYKYKVVKIEPLGVAPTRCKRRVVGRRRRRRAVGIGAVFLGFLGAAGSTMGAASMTLTVQA<br>RNLLSGNPDWLPDMTVWGIKQLQARVLAVERYLRDQQLLGIWGCSGKLICCTNVPWNSSWSN<br>RNLSEIWDNMTWLQWDKEISNYTQIIYGLLEESQNQQEKNEQDLLALDKWASLWNWFDITNW<br>LWYIRAGSGSGSGSGSASGDIIKLLNEQVNKEMQSSNLYMSMSSWCYTHSLDGAGLFLFDHAA<br>EEYEHAKKLIIFLNENNVPVQLTSISAPEHKFEGLTQIFQKAYEHEQHISESINNIVDHAIKSKDHA<br>TFNFLQWYVAEQHEEEVLFKDILDKIELIGNENHGLYLADQYVKGIAKSRKS

e. gp140 trimer-presenting LS and E2p nanoparticles

BG505 LS-10aa-gp140.664	MQIYEGKLTAEGLRFGIVASRFNHALVDRLVEGAIDCIVRHGGREEDITLVRVPGSWEIPVAAGE<br>LARKEDIDAVIAIGVLIRGATPHFDYIASEVSKGLANLALELRKPITFGVITADTLEQAIERAGTK<br>HGNKGWEAALSAIEMANLFKSLRGSGSGSGSGSASGAENLWVTVYYGVPVWKDAETTLFCAS<br>DAKAYETEKHNVWATHACVPTDPNPQEIHLENVTEEFNMWKNNMVEQMHTDIISLWDQSLKP<br>CVKLTPLCVTLQCTNVTNNITDDMRGELKNCSFNMTTELRDKKQKVYSLFYRLDVVQINENQG<br>NRSNNSNKEYRLINCNTSAITQACPKVSFEPIPIHYCAPAGFAILKCKDKKFNGTGPCPSVSTVQC<br>THGIKPVVSTQLLLNGSLAEEEVMIRSENITNNAKNILVQFNTPVQINCTRPNNNTRKSIRIGPGQ<br>AFYATGDIIGDIRQAHCNVSKATWNETLGKVVKQLRKHFGNNTIIRFANSSGGDLEVTTHSFNC<br>GGEFFYCNTSGLFNSTWISNTSVQGSNSTGSNDSITLPCRIKQIINMWQRIGQAMYAPPIQGVIRC<br>VSNITGLILTRDGGSTNSTTETFRPGGGDMRDNWRSELYKYKVVKIEPLGVAPTRCKRRVVGR<br>RRRRRAVGIGAVFLGFLGAAGSTMGAASMTLTVQARNLLSGNPDWLPDMTVWGIKQLQARV<br>LAVERYLRDQQLLGIWGCSGKLICCTNVPWNSSWSNRNLSEIWDNMTWLQWDKEISNYTQIIY

	GLLEESQNQQEKNEQDLLALD
BG505 gp140.664-E2p	AENLWVTVYYGVPVWKDAETTLFCASDAKAYETEKHNVWATHACVPTDPNPQEIHLENVTEE<br>FNMWKNNMVEQMHTDIISLWDQSLKPCVKLTPLCVTLQCTNVTNNITDDMRGELKNCSFNMT<br>TELRDKKQKVYSLFYRLDVVQINENQGNRSNNSNKEYRLINCNTSAITQACPKVSFEPIPIHYCA<br>PAGFAILKCKDKKFNGTGPCPSVSTVQCTHGIKPVVSTQLLLNGSLAEEEVMIRSENITNNAKNI<br>LVQFNTPVQINCTRPNNNTRKSIRIGPGQAFYATGDIIGDIRQAHCNVSKATWNETLGKVVKQL<br>RKHFGNNTIIRFANSSGGDLEVTTHSFNCGGEFFYCNTSGLFNSTWISNTSVQGSNSTGSNDSITL<br>PCRIKQIINMWQRIGQAMYAPPIQGVIRCVSNITGLILTRDGGSTNSTTETFRPGGGDMRDNWRS<br>ELYKYKVVKIEPLGVAPTRCKRRVVGRRRRRRAVGIGAVFLGFLGAAGSTMGAASMTLTVQA<br>RNLLSGNPDWLPDMTVWGIKQLQARVLAVERYLRDQQLLGIWGCSGKLICCTNVPWNSSWSN<br>RNLSEIWDNMTWLQWDKEISNYTQIIYGLLEESQNQQEKNEQDLLALD ASGAAAKPATTEGEF<br>PETREKMSGIRRAIAKAMVHSKHTAPHVTLMDEADVTKLVAHRKKFKAIAAEKGIKLTFLPYV<br>VKALVSALREYPVLNTAIDDETEEIIQKHYYNIGIAADTDRGLLVPVIKHADRKPIFALAQEINEL<br>AEKARDGKLTPGEMKGASCTITNIGSAGGQWFTPVINHPEVAILGIGRIAEKPIVRDGEIVAAPM<br>LALSLSFDHRMIDGATAQKALNHIKRLLSDPELLLM

[a] For each construct, the HIV-1 antigen is highlighted in gray shade with the mutations colored in orange. Mutations in the nanoparticle sequence designed to remove N-linked glycosylation sites are colored in green. The enzymatic site (ASG) between HIV-1 antigen and particle subunit is colored in magenta.

[b] All gp140 sequences contain a redesigned heptad repeat 1 (HR1) region that has been found to significantly improve trimer yield and purity while retaining the SOSIP-like structure and antigenicity (see the companion paper by Kong et al.). The modified HR1 region is highlighted in cyan shade and the 10-residue GS linker is colored in blue. A leader sequence “MDAMKRGLCCVLLLCGAVFVSPSQEIHARFRRGAR” is used for all gp140 nanoparticle constructs.
